# ANT and AIL6: masters of the master regulators during flower development

**DOI:** 10.1093/jxb/erab235

**Published:** 2021-07-28

**Authors:** Ángela G Juárez-Corona, Stefan de Folter

**Affiliations:** Unidad de Genómica Avanzada (UGA-Langebio), Centro de Investigación y de Estudios Avanzados del Instituto Politécnico Nacional (CINVESTAV-IPN), CP 36824 Irapuato, Guanajuato, México, USA

**Keywords:** AIL6, AINTEGUMENTA, ANT, flower development, growth, transcription factor

## Abstract

This article comments on:

**Krizek BA, Bantle AT, Heflin JM, Han H, Freese NH, Loraine AE**. 2021. AINTEGUMENTA and AINTEGUMENTA-LIKE6 directly regulate floral homeotic, growth, and vascular development genes in young Arabidopsis flowers. Journal of Experimental Botany **72**, 5478–5493.

**The transcription factor AINTEGUMENTA (ANT) and its paralog AIL6, are important for early flower development. Knowledge of their direct target genes gives us a better understanding of this process. Using a genome-wide ChIP-seq study, Krizek *et al.* (2021) investigated the DNA regions physically bound by the ANT or AIL6 protein in Arabidopsis. One of the key findings is that they directly regulate floral homeotic genes and genes related to growth and vasculature development in young flowers.**

Flower development is crucial for life on earth. Because of this, the mysteries of flower development have been studied for hundreds of years. In the last three decades, molecular biology has brought us into the era of gene cloning and genomics. Genome-wide expression studies and the identification of genome-wide transcription factor-binding sites allow us to define the gene regulatory networks (GRNs) that guide flower development. For Arabidopsis, several GRNs related to flower development have been reported (e.g. Pajoro *et al.*, 2014; Chen *et al.*, 2018; Zúñiga-Mayo *et al.*, 2019). Most of these GRNs are centered around one or several transcription factors.

ChIP for a specific transcription factor combined with next-generation sequencing (ChIP-seq) identifies protein–DNA binding events. ChIP-seq in combination with RNA-seq, using a mutant or an inducible system to activate the transcription factor of interest, is a powerful strategy to identify direct target genes that are regulated by the transcription factor of interest. Target genes that are regulated by the transcription factor can often be as few as 20% of the total genes bound by the transcription factor (Marsch-Martínez *et al.*, 2011). To date, this approach has been used for ~15 transcription factors involved in flower development in Arabidopsis (Pajoro *et al.*, 2014; Chen *et al.*, 2018). These include the famous homeotic genes of the ABC model, which encode transcription factors that act in a combinatorial fashion to specify floral organ identity (Krizek and Fletcher, 2005).

Now this approach has been utilized for the transcription factor AINTEGUMENTA (ANT) and its paralog AINTEGUMENTA-LIKE6 (AIL6) during early flower development (Krizek *et al.*, 2021). ANT and AIL6 participate in the establishment of the flower primordia and later promote the continued development and identity of petals, stamens, and carpels (Box 1) (Elliott *et al.*, 1996; Klucher *et al.*, 1996; Krizek, 1999, 2009; Han and Krizek, 2016).

**Box 1. ANT and AIL6 belong to the AP2 transcription factor family**

The *AINTEGUMENTA* (*ANT*) gene was cloned 25 years ago and shown to encode an AP2 transcription factor. *ANT* was described as a gene required for the initiation of integument growth in Arabidopsis ovules (Elliott *et al.*, 1996; Klucher *et al.*, 1996). It has been shown that loss-of-function *ant* mutants exhibit a reduction in the size of floral organs, abnormal integument development, abnormal ovule development, and a reduction in the number of ovules per flower. Furthermore, ANT has a role in the initiation and maintenance of early floral primordia growth, and the promotion of the development and identity of petals, stamens, and carpels, as shown in the figure (Elliott *et al.*, 1996; Klucher *et al.*, 1996; Krizek, 1999). Ectopic expression of *ANT* under a constitutive promoter results in increased growth of floral organs; this phenotype is the opposite of that resulting from loss of ANT function (Krizek, 1999; Mizukami and Fischer, 2000). This increased growth of floral organs is manifested as increased cell division; in contrast, the decreased size of *ant* mutant floral organs results from changes in the number and orientations of cell divisions within developing floral organ primordia (Krizek, 1999; Mizukami and Fischer, 2000).

There are seven AINTEGUMENTA-like (AIL)/PLETHORA (PLT) proteins that share high sequence similarity to ANT within the AP2 DNA-binding domain (Nole-Wilson *et al.*, 2005). AIL6/PLT3 acts redundantly with ANT during flower development (Krizek, 2009). The *ant ail6* double mutant shows defects during both vegetative and reproductive development. During reproductive development, its flowers exhibit altered positioning of floral organ primordia, loss of floral organ identity, and reduced growth of floral organ primordia (Krizek, 2009). Loss of AIL6 function on its own has no phenotypic consequences, indicating that all of its roles in flower development can be provided by *ANT* or some other gene. However, lines that express *AIL6* at higher levels display dosage-dependent phenotypes that include larger flowers, delayed cellular differentiation, a reduced number of floral organs, and the production of mosaic floral organs (Krizek and Eaddy, 2012; Han and Krizek, 2016). In summary, ANT has overlapping functions with AIL6 in flower development, including floral organ initiation, identity specification, growth, and patterning.

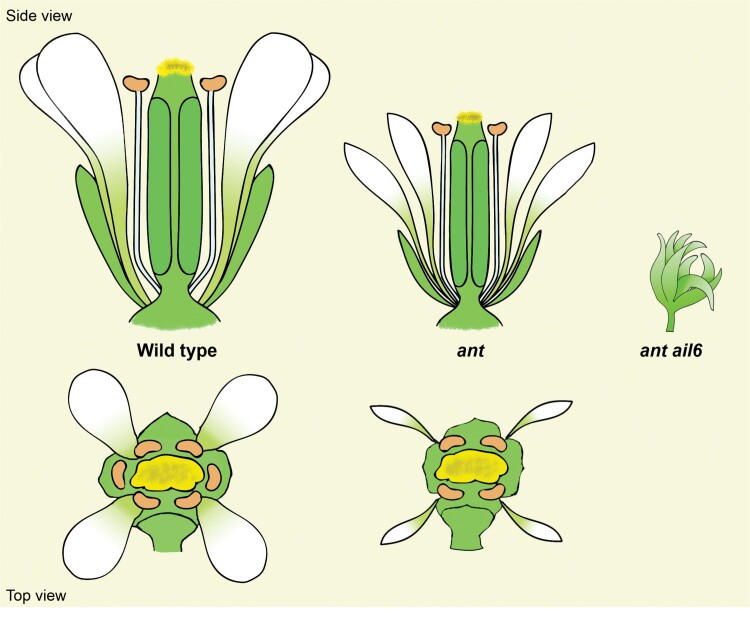

## ANT and AIL6 target genes

In their study, Krizek *et al.* (2021) report on the results of independent ChIP-seq experiments performed for both ANT and AIL6. Each gene was driven under its native promoter and tagged with the fluorescent marker VENUS, and introduced into a floral synchronization system, which results in inflorescences composed of flowers of a single stage of flower development (Ó’Maoiléidigh *et al.*, 2015). Moreover, for *ANT*, the mutant background was used. For the ChIP-seq experiments, floral stage 3 was used, which is the early developmental stage when sepal primordia become visible. Analyses of the data revealed for ANT a total of 2631 peaks associated with 2318 unique genes, while for AIL6, 595 peaks associated with 592 unique genes were found. Around 98% of the binding peaks of AIL6 overlap at least 50% with those of ANT. AIL6 is largely redundant to the ANT function (Krizek, 2009), which is nicely visible in the large overlap of binding peaks. It also has been reported that ANT has the more important role compared with AIL6 in floral organ development (Krizek, 2009). In line with this, the results clearly demonstrate a higher number of binding peaks detected for ANT in this ChIP-seq study (Krizek *et al.*, 2021). However, as the authors explain, it cannot be completely excluded that the reduced number of peaks detected for AIL6 may in part be due to technical issues, because the AIL6–VENUS translational fusion was not in the *ail6* mutant background and this could have resulted in competition with the endogenous protein during the ChIP part of the experiment. However, it is expected that ANT has more targets due to its more important role.

Among the shared targets bound by both ANT and AIL6 are genes involved in regulating many different developmental processes including polarity specification of the adaxial/abaxial axis, floral meristem determinacy, radial pattern formation, cell fate specification, meristem initiation, maintenance of meristem identity, plant ovule development, regulation of flower development, auxin-activated signaling pathway, and transcriptional regulation. ANT peaks not shared with AIL6 are related to genes involved in floral organ formation, stomatal complex morphogenesis, anther development, and leaf morphogenesis. Furthermore, various genes related to hormonal pathways such as the cytokinin-activated signaling pathway, auxin polar transport, response to gibberellin, ethylene-activated signaling pathway, and abscisic acid were observed.

In their study, Krizek *et al.* (2021) also report on DNA-binding motifs identified in the detected peaks. Based on previously determined ANT-binding motifs by SELEX (Nole-Wilson and Krizek, 2000; Krizek *et al.*, 2020) and AIL/PLT-binding motifs by DAP-seq (O’Malley *et al.*, 2016), the MEME software found ANT- and AIL6-binding motifs in ChIP-seq binding peaks. Furthermore, a significant overlap was observed between the ANT and AIL6 motifs, which suggests that ANT and AIL6 can both bind to this motif. Interestingly, binding motifs were also found for BASIC PENTACYSTEINE (BPC) and basic helix–loop–helix (bHLH) transcription factor proteins. In the ANT peaks, DNA motifs for basic leucine zipper transcription factors (bZIPs) were found.

RNA-seq information was used to determine which identified bound targets are also transcriptionally regulated by ANT and AIL6 (Krizek *et al.*, 2021). Based on expression data from the *ant ail6* double mutant (Krizek *et al.*, 2016), 29% of the shared targets of ANT and AIL6 are differentially expressed. Based on expression data from the inducible *35S:ANT-GR* line (Krizek *et al.*, 2020), 18% of the shared targets of ANT and AIL6 are differentially expressed. These numbers are similar to those found for other ChIP-seq studies (Marsch-Martínez *et al.*, 2011).

## Regulating master regulators

Interestingly, among the identified targets that are bound and regulated by ANT and AIL6 are three floral homeotic genes, *APETALA3* (*AP3*), *PISTILLATA* (*PI*), and *AGAMOUS* (*AG*) (Krizek *et al.*, 2021), which are often referred to as the master regulators of flower development. AP3 and PI specify petal and stamen identity, which is the B class function in the ABC model, and AG specifies stamen and carpel identity, which is the C class function (Krizek and Fletcher, 2005). All these tissues are altered in the *ant ail6* double mutant (Krizek, 2009) while petals and carpels show altered development in the inducible *AIL6* silencing line in the *ant* mutant background (Krizek *et al.*, 2021).

Autoregulation of expression is commonly seen for transcription factors, including the master regulators of the ABC model (de Folter and Angenent, 2006). This is also seen for ANT and AIL6 (Krizek *et al.*, 2021).

Other direct targets identified in the study are genes that regulate organ growth (Krizek *et al.*, 2021). Previous work has shown that altering ANT and AIL6 function results in reductions in floral organ size (Box 1). The identified targets include the growth repressor *BIG BROTHER* (*BB*), and the growth-promoting genes *ROTUNDIFOLIA3* (*ROT3*), *ANGUSTIFOLIA3*/*GRF-INTERACTING FACTOR 1* (*AN3*/*GIF1*), and *XYLOGLUCAN ENDOTRANSGLUCOSYLASE*/*HYDROLASE9* (*XTH9*).

Among the shared targets are genes with a role in vasculature development (Krizek *et al.*, 2021). Alterations in leaf vasculature pattern have been reported for *ant* and are more severe in the *ant ail6* double mutant (Kang *et al.*, 2007; Krizek, 2009). The identified target genes are *ERECTA-LIKE1* (*ERL1*), *PHLOEM INTERCALATED WITH XYLEM*/*TDIF RECEPTOR* (*PXY*/*TDR*), *CLAVATA3*/*ESR-RELATED 42* (*CLE42*), *MONOPTEROS* (*MP*), *TARGET OF MONOPTEROS 6* (*TMO6*), and *REVOLUTA* (*REV*).

## What is next?

In addition to many years of genetic work, the ChIP-seq studies of ANT and AIL6 (Krizek *et al.*, 2021) clearly demonstrate that ANT and AIL6 are important transcription factors for early flower development in Arabidopsis. In the current study, the authors used floral tissues in stage 3 of flower development. Recently, the Krizek group has published another ChIP-seq study of ANT, using floral tissues in stage 6/7 of flower development (Krizek *et al.*, 2020). This now provides the opportunity to study the dynamics of ANT targets during early flower development. As the authors briefly mentioned, some of the targets identified here are bound by ANT at a later floral stage, but there are also differences among the targets as flower development proceeds. Furthermore, comparisons with targets found in ChIP-seq studies of other floral regulators will be of interest (Pajoro *et al.*, 2014; Chen *et al.*, 2018). All of this new information will bring us closer to a more profound understanding of the gene regulatory network that orchestrates the complex process of early flower development.
